# Medicare Transitional Care Management Program and Changes in Timely Postdischarge Follow-Up

**DOI:** 10.1001/jamahealthforum.2024.0417

**Published:** 2024-04-12

**Authors:** Timothy S. Anderson, Shoshana J. Herzig, Edward R. Marcantonio, Robert W. Yeh, Jeffrey Souza, Bruce E. Landon

**Affiliations:** 1 Department of Medicine, Division of General Internal Medicine, University of Pittsburgh, Pittsburgh, Pennsylvania; 2 Division of General Medicine, Beth Israel Deaconess Medical Center, Boston, Massachusetts; 3 Harvard Medical School, Boston, Massachusetts; 4 Division of Cardiology, Beth Israel Deaconess Medical Center, Boston, Massachusetts; 5 Department of Health Care Policy, Harvard Medical School, Boston, Massachusetts

## Abstract

**Importance:**

In 2013, Medicare implemented payments for transitional care management (TCM) services, which provide increased reimbursement to clinicians providing ambulatory care to patients after discharge from medical facilities to the community.

**Objective:**

To determine whether the introduction of TCM payments was associated with an increase in timely postdischarge follow-up.

**Design, Setting, and Participants:**

This cross-sectional interrupted time-series study assessed quarterly postdischarge visit rates before (2010-2012) and after (2013-2019) TCM implementation 100% sample of Medicare fee-for-service beneficiaries discharged to the community after a hospital or skilled nursing facility stay. Data analyses were performed February 1 to December 15, 2023.

**Exposure:**

Implementation of payments for TCM.

**Main Outcomes and Measures:**

Timely postdischarge primary care follow-up, defined as receipt of a primary care ambulatory visit within 14 days of discharge. Secondary outcomes included receipt of a TCM visit and specialty care follow-up.

**Results:**

The study sample comprised 79 125 965 eligible discharges. Of these, 55.4% were female; 1.5% were Asian, 12.1% Black, 5.6% Hispanic, and 79.0% were White individuals; and 79.6% were beneficiaries aged 65 years and older. Timely primary care follow-up increased from 31.5% in 2010 to 38.8% in 2019 (absolute increase 7.3%), whereas specialist follow-up increased from 27.6% to 30.8% (absolute increase 3.2%). By 2019, 11.3% of eligible patients received TCM services. Interrupted time-series analyses demonstrated an increased slope of timely primary care follow-up after the introduction of TCM services (pre-TCM slope, 0.12% per quarter vs post-TCM slope, 0.29% per quarter; difference, 0.13%; 95% CI, 0.02% to 0.22%). Receipt of timely follow-up increased for all demographic groups; however, Black, Hispanic, and Medicaid dual-eligible patients and patients residing in urban areas and counties with high-level social deprivation were less likely to receive follow-up during the study period. These disparities widened for Black patients (difference-in-differences in pre-TCM vs post-TCM slope, −0.14%; 95% CI, −0.25% to −0.2%) and patients who were Medicaid dual-eligible (difference-in-differences pre-TCM vs post-TCM slope, −0.21%; 95% CI, −0.35% to −0.07%).

**Conclusions:**

These findings indicate that Medicare’s introduction of payments for TCM services was associated with a persistent increase in the rate of timely postdischarge primary care but did not narrow demographic or socioeconomic disparities. Most beneficiaries did not receive timely primary care follow-up.

## Key Points

**Question:**

Has Medicare’s implementation of payments for transitional care management (TCM) services been associated with an increase in timely primary care follow-up after medical facility discharge?

**Findings:**

In this cross-sectional interrupted time-series study of 77 million Medicare discharges from 2010 to 2019, implementation of TCM payments was associated with a statistically significant and persistent increase in rates of postdischarge primary care follow-up, increasing from 31.9% in 2010 to 39.2% in 2019. Despite this increase, demographic and socioeconomic disparities in rates of follow-up persisted.

**Meaning:**

These findings indicate that the implementation of TCM payments was associated with an increase in the rate of timely primary care follow-up after discharge; however, most beneficiaries did not receive timely follow-up care.

## Teaser

This cross-sectional analysis assesses whether the introduction of Transitional Care Management payments by Medicare has been associated with an increased timely postdischarge follow-up.

## Introduction

The transition home after a hospitalization is a high-risk period requiring substantial coordination of care.^1,2^ At discharge, patients are frequently instructed to coordinate multiple specialist visits, follow-up testing, medication changes, and rehabilitation programs, all while recovering from the functional and cognitive impairments that often follow an acute illness or surgery. In 2013, Medicare adopted a novel payment program for clinicians managing patients’ transition home after discharge from hospitals or nursing homes.^3^ This transitional care management (TCM) payment offers increased compensation of approximately 130% to 150% compared with typical office visits for clinicians who provide a communication with the patient by the clinician or a designated clinical staff member within 2 business days of discharge, an office visit within 14 days of discharge, a medication reconciliation that can occur during the phone call or office visit, and ongoing care coordination, eg, coordination of primary care and specialist visits.

Medicare spending on TCM services between 2013 and 2018 was nearly $1 billion,^4^ yet outcomes of the TCM program have not been well characterized. Descriptive studies of the first 4 years of the program have shown that TCM services were delivered to fewer than 10% of eligible patients^5^ and were highly concentrated among a small number of practices.^6,7^ One of these studies found that patients who received TCM services in the first month after discharge had reduced costs and mortality in the second month after discharge compared with patients who did not receive TCM.^6^

A key unanswered question is whether TCM has led to increased rates of postdischarge follow-up or whether TCM billing has been substituted for evaluation and management office visits. Furthermore, the equity of TCM delivery has not been evaluated. If delivered equitably, TCM services have the potential to reduce known disparities in postdischarge outcomes across multiple domains of the social determinants of health.^8,9,10,11^ Whether there are disparities in postdischarge follow-up and how the TCM program has affected diverse demographic groups is unknown. This study of all Medicare fee-for-service beneficiaries between 2010 and 2019 sought to evaluate whether the introduction of TCM services was associated with an increased rate of timely postdischarge follow-up and whether changes in follow-up differentially affected sociodemographic groups.

## Methods

This project was approved by the CMS privacy board and the Harvard Medical School institutional review committee, which also waived the requirement for obtaining informed consent because all administrative claims data were deidentified. The study followed the Strengthening the Reporting of Observational Studies in Epidemiology (STROBE) reporting guideline.

### Data Collection and Participants

We analyzed administrative inpatient and outpatient claims from the US Centers for Medicare & Medicaid Services (CMS) database for all traditional Medicare beneficiaries from 2010 to 2020 (100% files). The cohort included hospitalizations and observation stays with a discharge date between January 1, 2010, and December 31, 2019, of beneficiaries who were continuously enrolled in Medicare Parts A and B in the 12 months prior to index hospitalization and 1 month after discharge (or until death if sooner). We excluded beneficiaries enrolled in a Medicare Advantage plan during this same period because their outpatient claims were not available. Hospitalizations ending in death or discharge to hospice or another acute or long-term care hospital were excluded.

Hospitalizations that ended with discharge to skilled nursing facilities were identified by overlapping claims (nursing home claim the same day or day following hospital discharge date) and were included if beneficiaries were subsequently discharged to the community from the skilled nursing facility. For these episodes, the index date was the date of skilled nursing home discharge. The unit of analysis was the facility stay; thus, beneficiaries could contribute multiple occurrences.

### Outcomes

The primary outcome was receipt of a primary care ambulatory visit within 14 days of discharge (hereafter, timely primary care follow-up). The 14-day time period was chosen based on the CMS requirement that TCM visits must be delivered within 14 days of discharge.^3^ Ambulatory visits included TCM visits and non-TCM evaluation and management or preventive visits identified by *Current Procedural Technology* codes (eTable 1 in Supplement 1). Primary care visits included visits billed by physicians with specialties of general practice, family practice, internal medicine, osteopathic manipulative therapy, geriatrics, and preventive medicine. As Medicare does not report the specialty of advanced practice clinicians, we classified nurse practitioners and physician assistants who shared the same practice taxpayer identification number (TIN) as primary care clinicians.

Secondary outcomes included receipt of ambulatory visits from a specialist clinician, receipt of ambulatory visits from any clinician within 14 days of discharge, and receipt of each outcome within 30 days of discharge. We examined rates of specialist clinician visits as the TCM program was primarily intended for primary care clinics,^3^ but recent research has suggested that TCM billing by specialists is common.^7^

### Patient Measures

Given known disparities in postdischarge outcomes across demographic and socioeconomic groups,^8,9,10,11^ we sought to describe disparities in postdischarge follow-up and assess the association of the TCM program with existing disparities. We assessed follow-up for 5 demographic subgroups: sex, race and ethnicity, Medicaid dual-eligibility, county-level social deprivation index (SDI), and rurality. Race and ethnicity data were collected from the Medicare Beneficiary Summary File using the Research Triangle Institute race code and grouped as Asian, Black, Hispanic, White, or other (American Indian/Alaska Native, unknown, and any other race/ethnicity). SDI is a composite measure of 7 demographic characteristics collected in the American Community Survey which have been associated with poor health care access.^12,13^ The SDI score ranges from 1 to 100 and was examined in quartiles, with quartile 1 (SDI score 1-25) reflecting the least disadvantaged counties. Residence area was defined based on the 2010 rural-urban commuting area (RUCA) codes.^14,15^

We additionally examined whether postdischarge follow-up differed for patients attributed to accountable care organizations (ACOs) using Medicare Shared Savings Program attribution rules vs those who were not attributed to an ACO.^16^ We hypothesized that ACOs face greater financial incentives to reduce readmissions and ACOs that deliver both inpatient and outpatient services may be best aligned to deliver timely follow-up. Then we evaluated whether patients who were more connected to primary care, defined as receiving a visit in the year prior to hospital admission, had higher rates of postdischarge follow-up given evidence of longer wait times for new patient visits.^17^

### Statistical Analysis

We conducted an interrupted time-series (ITS) analysis with the introduction of the TCM program as the intervention. Our analysis included all eligible hospital discharges from 2010 to 2012 (pre-TCM period) and 2016 to 2019 (post-TCM period). We treated 2013 to 2015 as an interruption given early confusion over the timing of billing TCM visits (at the date of visit vs day 30 after discharge) during their initial role out.^18^ Quarterly outcomes were first assessed graphically and then assessed using ordinary least squares regression with Newey-West standard errors, to account for autocorrelation.^19^ Predicted rates for the primary outcome were estimated assuming the presence and absence of the intervention for the end of the study period (December 2019) using postestimation linear combination equations.^20^ Similar models were run for 30-day outcomes but using 2013 to 2019 as the post-TCM period.

To assess the how the TCM program affected demographic disparities in outpatient follow-up, we conducted similar comparative ITS analyses,^21^ estimating separate models for each demographic characteristic with the addition of a categorical variable identifying the demographic covariate and interaction terms for this covariate, quarter, and intervention period. For analyses of race and ethnicity groups, we compared Asian, Black, and Hispanic populations with White as the reference population, and excluded other racial groups from this analysis because of lower validity of imputed race codes and smaller sample sizes.^22,23^ Data analyses were performed February 1 to December 15, 2023, using Stata, release 17.0 (StataCorp LLC), with statistical significance set at 2-sided α = .05.

## Results

There were 79 125 965 eligible discharges between January 1, 2010, and December 31, 2019, of which 77.9% were discharges from acute hospitalizations and 22.1% were from skilled nursing facilities. Of the total study population 20.4% were younger than 65 years and 20.2% were 85 years or older; 55.4% were female and 44.6%, male; 1.5% were Asian, 12.1% Black, 5.6% Hispanic, and 79.0% White individuals; and 32.5% were Medicaid eligible. Baseline patient demographic characteristics were similar in the 2 study periods (Table 1).

**Table 1.** Demographics Characteristics of Study Population of Medicare Beneficiaries, Pre- and Post-TCM, 2010 to 2019

Characteristic	%	
	Pre-TCM (2010-2012)	Post-TCM (2013-2019)
Total beneficiaries, No.	25 586 725	53 539 240
Age group, y		
<65	20.7	20.3
65-69	12.2	13.3
70-74	15.6	16.7
75-79	15.5	15.7
80-84	15.4	13.9
≥85	20.6	20.0
Sex		
Female	56.3	54.9
Male	43.7	45.1
Race and ethnicity		
Asian	1.4	1.6
Black	12.3	11.9
Hispanic	5.5	5.6
White	79.3	78.8
Other^a^	1.3	2.0
US region		
Midwest	25.2	24.1
Northeast	19.3	19.2
South	41.0	41.0
West	14.6	15.8
Medicaid dual-eligibility		
Dual-eligible	33.9	31.9
Not dual-eligible	66.1	68.1
Residence area^b^		
Rural	23.2	22.8
Urban	76.8	77.2
ACO attribution		
Attributed to ACO	0.8	45.2
Not attributed to ACO	99.2	54.8
PCP visit in prior year		
Any	82.0	81.2
None	18.0	18.8
County-level SDI score^b^		
1-25 (Least disadvantaged)	25.0	26.2
26-50	25.5	25.8
51-75	26.4	26.1
76-100 (Most disadvantaged)	23.0	21.8
Disposition at hospital discharge		
Home	57.6	54.8
Home health care	20.1	22.4
SNF	22.3	22.8
Length of stay, d		
<2	12.3	12.0
2-7	60.7	60.7
8-14	13.6	13.4
15-30	9.2	9.7
>30	4.2	4.3

Abbreviations: ACO, Accountable Care Organization; PCP, primary care practitioner; SDI, social deprivation index; SNF, skilled nursing facility; TCM, Transitional Care Management.

^a^ This category included American Indian or Alaska Native, unknown, and any other race or ethnicity.

^b^ Missing residence (<0.02%) and SDI (<0.1%) data in both periods.

**Figure 1.**
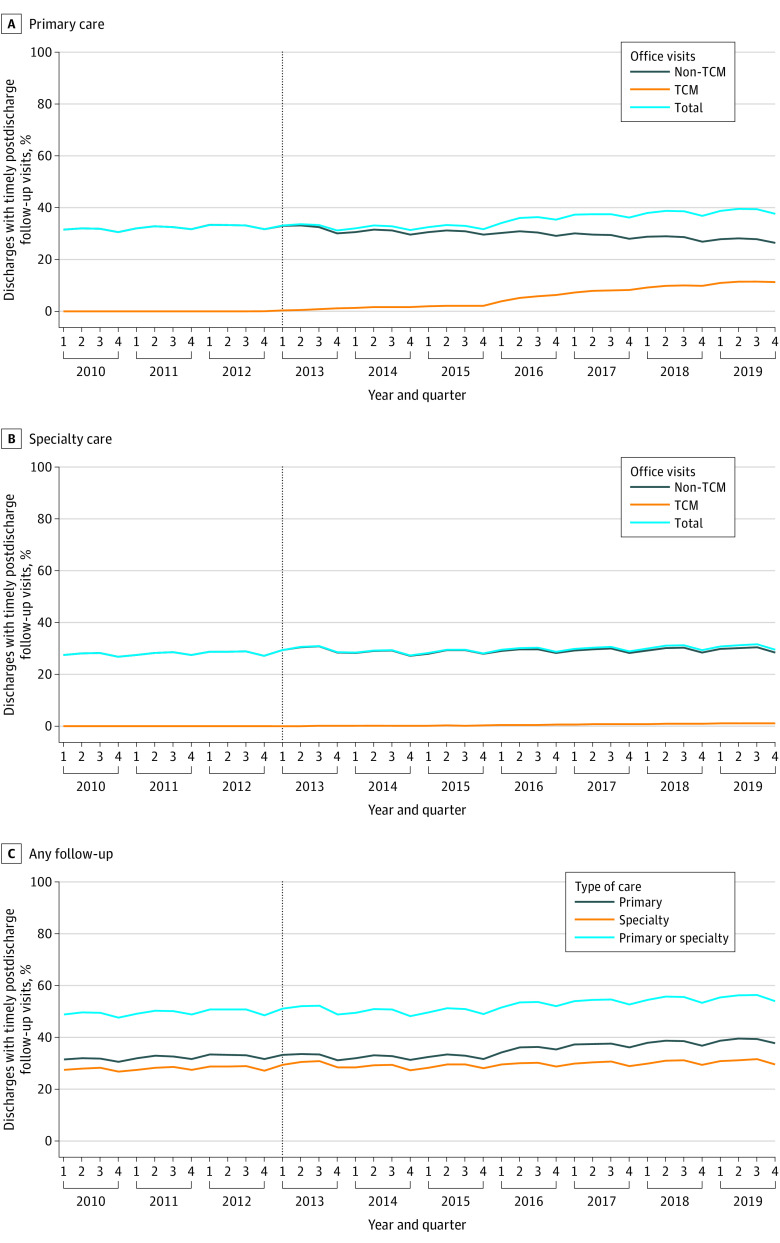
Trends in Timely Postdischarge Follow-Up, 2010 to 2019 Total refers to receipt of any ambulatory visit, Transitional Care Management (TCM), and/or non-TCM visit, within 14 days of discharge. Office visit refers to patients who received an office visit without a TCM billing code; patients who received both TCM and non-TCM office visits within 14 days were grouped in the TCM category.

Trends in timely primary care and specialty care follow-up are shown in Figure 1 and Table 2. In 2010, 31.5% of patients received timely primary care follow-up and this increased to 38.8% in 2019 (absolute increase, 7.3%). Provision of TCM services increased throughout the study period and by 2019, 11.3% of eligible patients received primary care TCM services whereas the proportion of patients receiving non-TCM primary care office visits declined from 31.5% to 27.5% (absolute change, −3.5%). Changes in postdischarge specialist visit rates were more modest, increasing from 27.6% in 2010 to 30.8% in 2019 (absolute increase, 3.2%). By 2019, 55.5% of patients received timely primary care or specialty visits.

**Table 2.** Interrupted Time-Series Estimates of the Association Between Transitional Care Management (TCM) and Timely Postdischarge Follow-Up

Care type	Outcome, %			Quarterly % change (95% CI)		
	2010	2019	Absolute change	Pre-TCM slope	Post-TCM slope	Absolute change in slope
Primary care						
Timely follow-up	31.5	38.8	7.3	0.12 (0.07 to 0.17)^a^	0.25 (0.18 to 0.31)^a^	0.13 (0.03 to 0.22)^a^
TCM	0	11.2	11.3	NA	NA	NA
Office visit	31.5	27.5	−3.9	0.12 (0.07 to 0.17)^a^	−0.18 (−0.21 to −0.16)^a^	−0.30 (−0.37 to −0.24)^a^
Specialty care	27.6	30.8	3.2	0.06 (0.01 to 0.11)^a^	0.08 (0.05 to 0.11)^a^	0.02 (−0.04 to 0.07)
Primary or specialty care	48.9	55.5	6.6	0.10 (0.03 to 0.17)^a^	0.21 (0.15 to 0.26)^a^	0.10 (0.01 to 0.20)

Abbreviation. NA, not applicable.

^a^ Statistical significance based on 95% CIs.

### Interrupted Time-Series Analysis of TCM Introduction

ITS analyses demonstrated a significantly increased slope of timely primary care follow-up after the introduction of TCM services (pre-TCM slope, 0.12% per quarter vs post-TCM slope, 0.25% per quarter; difference, 0.13%; 95% CI, 0.03% to 0.22%) (Table 2). Had the pre-TCM slope continued, without the introduction of TCM services, 14-day primary care follow-up rates in 2019 were predicted to be 6.1 percentage points lower (95% CI, −8.7% to −3.5%). There was no difference in slope of timely specialist follow-up after the introduction of TCM services (pre-TCM vs post-TCM slope difference, 0.02%; 95% CI, −0.04% to 0.07%).

Absolute rates of 30-day primary care follow-up and 30-day specialist follow-up were higher than 14-day outcomes, but trends were similar. There was an increase in slope of 30-day primary care follow-up (pre-TCM vs post-TCM slope difference 0.12%; 95% CI, 0.05% to 0.19%), but not specialist follow-up (pre-TCM vs post-TCM slope difference, −0.004%; 95% CI, −0.08% to 0.07%) (eTable 2 in Supplement 1).

### Demographic Subgroups

**Figure 2.**
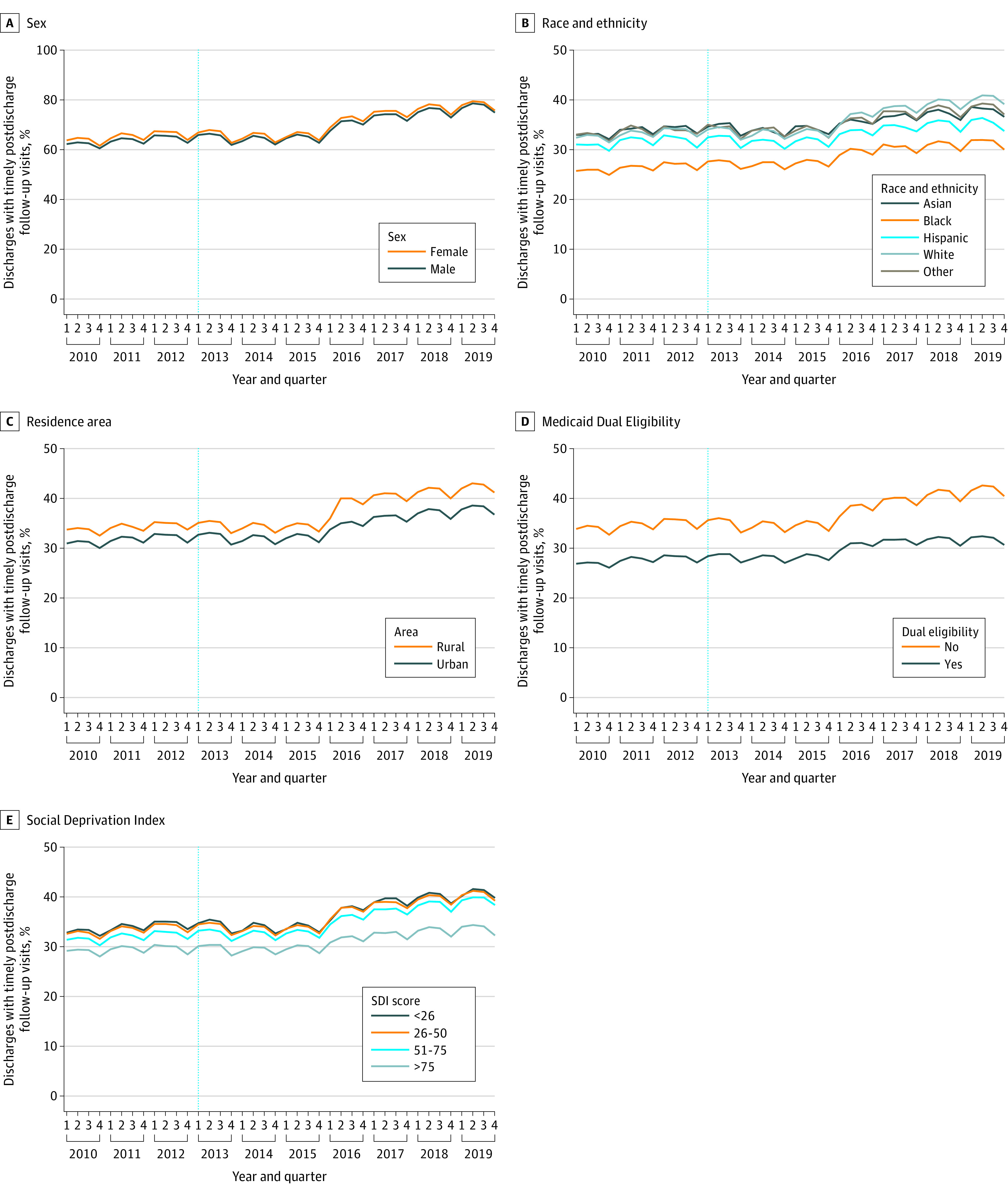
Trends in Timely Postdischarge Primary Care Follow-up, by Demographic and Socioeconomic Group Excludes discharges with missing residence (<0.02%) or SDI (<0.1%) data. Other included American Indian or Alaska Native, unknown, and any other race or ethnicity. SDI indicates Social Deprivation Index.

Trends in timely primary care follow-up for subgroups are shown in Figure 2. Across the study period, there were no differences in trends by sex (Figure 2A). In 2010, 25.6% of Black patients received timely follow-up compared with 32.8% of Asian, 30.7% of Hispanic, and 32.4% of White patients. Although all groups showed increased follow-up rates during the study period, by 2019, White patients experienced the highest follow-up rate (40.2%), whereas among Black patients, the follow-up rate increased to only 31.4%, a larger disparity than was observed in 2010 (6.8% difference in 2010 vs 8.7% difference in 2019; difference-in-differences pre- vs post-TCM slope, 0.14%; 95% CI, 0.02%-0.25%) (Figure 2B; eTable 3 in Supplement 1). There were no significant differences between Asian or Hispanic patients compared with White patients in slopes of timely primary care follow-up comparing periods.

Patients residing in rural areas were more likely to receive timely primary care follow-up throughout the study period. Although rates of follow-up increased for both groups, by 2019, 42.2% of rural-residing patients receiving timely primary care follow-up compared with 37.8% of urban-residing patients (absolute difference, 4.4%) but there were no significant difference-in-differences in slopes of timely primary care follow-up comparing periods (Figure 2C; Table 3).

**Table 3.** Interrupted Time-Series Estimates of the Association Between TCM Program Introduction and 14-Day Postdischarge Primary Care Follow-Up, by Demographic Characteristic

Characteristic	14-d Primary care follow-up						
	%			Quarterly % change (95% CI)			
	2010	2019	Absolute change	Pre-TCM slope	Post-TCM slope	Absolute change in slope	Difference in slope change
Sex							
Female	31.8	39.0	7.2	0.12 (0.06 to 0.18)^a^	0.24 (0.17 to 0.30)^a^	0.12 (0.02 to 0.22)^a^	−0.02 (−0.14 to 0.11)
Male	31.0	38.6	7.5	0.12 (0.07 to 0.17)^a^	0.26 (0.20 to 0.31)^a^	0.14 (0.05 to 0.22)^a^	0 [Reference]
Race and ethnicity							
Asian	32.8	37.9	5.1	0.14 (0.08 to 0.20)^a^	0.16 (0.11 to 0.21)^a^	0.02 (−0.07 to 0.10)	−0.06 (−0.65 to 0.54)
Black	25.6	31.4	5.8	0.12 (0.07 to 0.16)^a^	0.13 (0.08 to 0.17)^a^	0.01 (−0.06 to 0.08)	−0.14 (−0.25 to −0.02)^a^
Hispanic	30.7	35.4	4.7	0.09 (0.0004 to 0.18)^a^	0.12 (0.06 to 0.19)^a^	0.03 (−0.10 to 0.16)	−0.12 (−0.28 to 0.04)
White	32.4	40.2	7.8	0.13 (0.07 to 0.18)^a^	0.27 (0.21 to 0.34)^a^	0.15 (0.05 to 0.24)^a^	0 [Reference]
Residence area^b^							
Rural	33.5	42.2	8.7	0.10 (0.05 to 0.14)^a^	0.27 (0.19 to 0.35)^a^	0.17 (0.07 to 0.27)^a^	0.06 (−0.08 to 0.19)
Urban	30.9	37.8	7.0	0.13 (0.07 to 0.18)^a^	0.24 (0.18 to 0.30)^a^	0.11 (0.02 to 0.20)^a^	0 [Reference]
Medicaid dual eligibility							
Not eligible	33.8	41.8	7.9	0.12 (0.06 to 0.18)^a^	0.29 (0.23 to 0.36)^a^	0.17 (0.07 to 0.28)^a^	0 [Reference]
Dual eligible	26.8	31.9	5.0	0.12 (0.07 to 0.17)^a^	0.08 (0.02 to 0.15)^a^	−0.04 (−0.13 to 0.06)	−0.21 (−0.35 to −0.07)^a^
County-level SDI score^b^							
1-25	32.9	40.7	7.8	0.17 (0.12 to 0.22)^a^	0.27 (0.19 to 0.36)^a^	0.11 (−0.002 to 0.21)	0 [Reference]
26-50	32.5	40.4	7.9	0.14 (0.08 to 0.20)^a^	0.25 (0.20 to 0.31)^a^	0.11 (0.02 to 0.21)^a^	0.01 (−0.14 to 0.15)
51-75	31.3	39.4	8.1	0.11 (0.07 to 0.16)^a^	0.28 (0.23 to 0.33)^a^	0.16 (0.09 to 0.24)^a^	0.06 (−0.08 to 0.19)
76-100	29.0	33.7	4.7	0.04 (−0.01 to 0.10)	0.15 (0.10 to 0.20)^a^	0.11 (0.03 to 0.19)^a^	0.003 (−0.14 to 0.14)

Abbreviations: SDI, social deprivation index; TCM, Transitional Care Management.

^a^ Statistical significance based on 95% CIs.

^b^ Missing residence (<0.02%) and SDI (<0.1%) data in both periods.

### Socioeconomic Subgroups

Patients who were dual-eligible for Medicaid were less likely to receive timely primary care follow-up throughout the study period (Figure 2D; Table 3). Although rates of follow-up increased for dual-eligible and nondual-eligible populations, by 2019 the disparity between groups had increased, with 32.2% of patients who were dual-eligible receiving timely primary care follow-up compared with 42.2% of patients who were not dual-eligible (absolute difference, 9.9%). Comparative ITS analysis demonstrated a significantly greater change in slope TCM introduction for patients who were not dual-eligible compared with those dual eligibility (difference-in-differences pre- vs post-TCM slope, 0.21%; 95% CI, 0.07%-0.35%).

Patients residing in counties with the highest level of social deprivation had the lowest rates of timely primary care follow-up throughout the study period (eFigure 1 in Supplement 1; Table 3). This disparity widened overtime, with the difference in follow-up between highest and lowest SDI counties increasing from 4.0% in 2010 to 7.1% in 2019, although there were no significant differences in the change in slope after TCM introduction across groups. By 2019, rates of TCM delivery varied between subgroups in patterns similar to rate of timely primary care follow-up overall, with the lowest rates among Black, Hispanic, and Medicaid dual-eligible patients (eFigure 2 in Supplement 1).

### Differences by ACO and Primary Care Connectedness

Patients attributed to ACOs had higher rates of receipt of both timely primary care follow-up and TCM visits, which increased through the study period (eFigure 3 in Supplement 1). By 2019, patients attributed to ACOs had an absolute 5.2% greater rate of TCM visits and an absolute 5.2% greater rate of timely primary care follow-up.

Patients who had a primary care visit in the prior year also had substantially higher rates of receipt of both timely primary care follow-up and TCM visits throughout the study period. In 2010, the difference in timely primary care follow-up between patients with and without recent primary care was 28.2% (37.1% vs 8.9%), decreasing to 25.4% in 2019 (44.3% vs 19.0%). By 2019, patients with recent primary care had an absolute 8.5% greater rate of TCM visits (eFigure 4 in Supplement 1).

## Discussion

This national Medicare analysis found that the introduction of TCM payments was associated with a persistent increase in the rate of timely postdischarge primary care follow-up. Rates of timely primary care follow-up increased 7 percentage points between 2010 and 2019, with an increase in TCM visits comprising most of the total increase. Despite this, by 2019, 60% of patients still did not receive timely primary care follow-up and only 11% of eligible patients received TCM services. The introduction of TCM payments did not improve observed disparities in receipt of postdischarge follow-up, with inequalities across race and ethnicities, Medicaid dual-eligible status, and county-level social deprivation widening during the study period.

The observed increase in timely postdischarge primary care follow-up was likely driven in part by the financial incentive created by TCM billing codes, which provide approximately 130% to 150% of the reimbursement of a comparable evaluation and management visits,^24^ encouraging ambulatory clinicians to prioritize postdischarge visits. While rates of timely primary care follow-up were increasing before the introduction of TCM payments, the slope of growth increased in the post-TCM period (2016-2019). This association was not seen for specialist visits, suggesting that although TCM can be billed by any specialty, it has primarily been adopted by primary care as was intended by CMS.^3^

These findings may also be associated with other policy measures implemented during the same period. Medicare’s Hospital Readmissions Reduction Program (HRRP) was implemented in October 2012, 3 months before the TCM program.^25^ Although both programs have similar overarching goals to improve postdischarge outcomes, they use very different strategies, with TCM being squarely aimed at primary care clinicians and the HRRP, at providing incentives for hospitals at the time of discharge. TCM payments provide a positive financial incentive and target primary care clinicians, regardless of the reason for patients’ hospitalization, whereas the HRRP targets specific clinical conditions and was designed to financially penalize hospitals with higher than expected 30-day readmission rates. The effects of HRRP have been varied and the program itself is controversial, particularly with regard to penalizing hospitals that serve vulnerable populations.^26^ This combination of financial penalties and incentives from these 2 programs effectively shifts CMS payments from low-performing hospitals to ambulatory clinicians. The voluntary Medicare Shared Savings Program began in 2012, and financial incentives to reduce spending (including readmissions) for ACOs also may have been associated with increased focus on improving care transitions.^27^ We observed higher rates of TCM delivery and of timely postdischarge follow-up among patients attributed to ACOs, and these rates of follow-up were higher throughout the follow-up period. Through integration of inpatient and outpatient clinicians and systems, ACOs may have the infrastructure to deliver TCM, which requires identification of discharges patients to deliver the required outreach telephone call within 2 business days of discharge, a requirement that may also contribute to lower rates of TCM delivery for less-resourced practices.

Irrespective of the introduction of increased payments for TCM services, we identified substantial disparities in timely posthospital follow-up across demographic groups, which persisted over time. This novel finding builds on the literature describing disparities in primary care access broadly^28,29^ and may contribute to documented disparities in posthospital outcomes for racial and ethnicity minorities and individuals residing in disadvantaged neighborhoods. However, despite substantial geographic challenges in accessing primary care, rural-residing patients were substantially more likely than urban-residing patients to receive timely follow-up, whereas posthospital mortality and readmission rates have been found to be higher for rural Medicare beneficiaries in prior studies.^10^

In theory, timely postdischarge primary care follow-up has numerous benefits, including coordination of the patient’s care, ensuring home safety, monitoring for delayed complications of hospitalizations, and follow-up of outstanding tests and treatments started during hospitalization. In practice, the necessary components of postdischarge follow-up and the magnitude of benefit are less certain.^30,31,32^ Although routine postdischarge follow-up may not be necessary for all patients, and in some cases specialist follow-up may be higher priority, given the frequency of adverse events and the need for care coordination in the postdischarge period, many patients likely benefit, particularly older Medicare beneficiaries with multiple morbidities. Increased posthospital follow-up is unlikely to be harmful; however, it does place cost, time, and transport burdens on patients for whom telehealth may offer a potential solution.^33^ In this study, we did not assess whether patients were rehospitalized or died during the 30-day follow-up period. Although early mortality or readmission may preclude patients from receiving timely follow-up, it may reduce the risk of subsequent negative outcomes; therefore, advanced causal inference methods are necessary to identify associations between these outcomes in future studies. Reduced rates of postdischarge readmission or mortality may explain some of the increase in postdischarge follow-up; however, prior descriptive studies indicate that changes in these outcomes during the study period were modest. Given that 30-day all-cause readmissions declined less than 1.5% from 2010 to 2016^34^ and 30-day postdischarge all-cause mortality declined less than 0.5% from 2010 to 2017 for beneficiaries aged 65 years or older,^35^ these are unlikely to fully explain the observed findings.

### Limitations

This study had several limitations. First, although our findings are consistent with the association of the TCM program with increased rates of timely postdischarge primary care follow-up, this study cannot prove causality. Patterns of care could be partly driven by other time-varying factors, including the introduction of the HRRP and the Medicare ACO programs as well as changes in patient populations enrolled in traditional Medicare compared with Medicare Advantage. Second, patients may receive unbilled follow-up, through telephone calls or electronic messaging not captured by the current study. Third, analyses of difference by race and ethnicity were limited by reliance on impute categories, which are less accurate for Asian and Hispanic patients.^22,23^ Fourth, we did not assess trends in postdischarge follow-up during the COVID-19 pandemic and the associated substantial disruptions in both inpatient and outpatient care delivery. However, other studies have indicated that there was substantial uptake of telehealth for the delivery of postdischarge ambulatory care during the early pandemic,^33^ which warrant further investigation given that TCM visits are telehealth-eligible. Lastly, future research is required to better understand how timely postdischarge follow-up affects clinical outcomes such as readmission rates and mortality.

## Conclusions

The findings of this cross-sectional study suggest that the introduction of TCM payments by Medicare was associated with a significant and persistent increase in the quarterly rate of timely postdischarge primary care follow-up. However, most patients did not receive timely follow-up care, and demographic disparities in timely follow-up rates did not narrow.

## Notes

Supplement 1. eTable 1. Ambulatory Visit Codes eTable 2. Interrupted Time Series Estimates of the Association between Transitional Care Management and 30-Day Post-Discharge Follow Up eTable 3. Interrupted Time Series Estimates of the Association between Transitional Care Management and 30-Day Post-Discharge Follow Up, by Demographics eFigure 1. Trends in 30-Day Post-Discharge Follow Up, 2010-2019 eFigure 2. Rates of Transitional Care Management Visits in 2019, by Demographics eFigure 3. Trends in Timely Post-Discharge Follow Up, 2010-2019, by ACO Attribution eFigure 4. Trends in Timely Post-Discharge Follow Up, 2010-2019, by Recent Primary Care

Supplement 2. Data Sharing Statement
