# Supplementary material for: Medicare Transitional Care Management Program and Changes in Timely Postdischarge Follow-Up
Source: JAMA Health Forum. 2024 Apr 12;5(4):e240417. doi: 10.1001/jamahealthforum.2024.0417 (PMC11065163; doi:10.1001/jamahealthforum.2024.0417)
Supplement: Supplement 1. — eTable 1. Ambulatory Visit Codes eTable 2. Interrupted Time Series Estimates of the Association between Transitional Care Management and 30-Day Post-Discharge Follow Up eTable 3. Interrupted Time Series Estimates of the Association between Transitional Care Management and 30-Day Post-Discharge Follow Up, by Demographics eFigure 1. Trends in 30-Day Post-Discharge Follow Up, 2010-2019 eFigure 2. Rates of Transitional Care Management Visits in 2019, by Demographics eFigure 3. Trends in Timely Post-Discharge Follow Up, 2010-2019, by ACO Attribution eFigure 4. Trends in Timely Post-Discharge Follow Up, 2010-2019, by Recent Primary Care [file jamahealthforum-e240417-s001.pdf]

Supplemental Online Content

Anderson TS, Herzig SJ, Marcantonio ER, Yeh RW, Souza J, Landon BE. Medicare Transitional Care Management Program and Changes in Timely Postdischarge Follow-Up. *JAMA Health Forum.* Published online April 12, 2024.
doi:10.1001/jamahealthforum.2024.0417

eTable 1. Ambulatory Visit Codes

eTable 2. Interrupted Time Series Estimates of the Association between Transitional Care Management and 30-Day Post-Discharge Follow Up

eTable 3. Interrupted Time Series Estimates of the Association between Transitional Care Management and 30-Day Post-Discharge Follow Up, by Demographics

eFigure 1. Trends in 30-Day Post-Discharge Follow Up, 2010-2019

eFigure 2. Rates of Transitional Care Management Visits in 2019, by Demographics

eFigure 3. Trends in Timely Post-Discharge Follow Up, 2010-2019, by ACO Attribution

eFigure 4. Trends in Timely Post-Discharge Follow Up, 2010-2019, by Recent Primary Care

This supplemental material has been provided by the authors to give readers additional information about their work.

© 2024 Anderson TS et al. *JAMA Health Forum.*

eTable 1: Ambulatory Visit Codes

Visit Type	CPT Codes
Transitional Care Management (TCM) Visit	99495 – 99496
**Non-TCM Ambulatory Visits**	
Office or Other Outpatient Visit for an Established Patient	99211 – 99215
New Patient Office Visit	99201 – 99205
Annual Wellness Visit	G0438, G0439

© 2024 Anderson TS et al. *JAMA Health Forum.*

eTable 2: Interrupted Time Series Estimates of the Association between Transitional Care Management and 30-Day Post-Discharge Follow Up

30-Day Follow Up	Outcome in 2010, %	Outcome in 2019, %	Absolute Change, %	Pre-TCM Slope, quarterly % change (95% CI)	Post-TCM Slope, quarterly % change (95% CI)	Absolute Change in Slope, quarterly % change (95% CI)
Primary Care						
Timely Follow Up	45.0	52.2	7.2	0.11 (0.05 – 0.17)*	0.23 (0.19 – 0.26)*	0.12 (0.05 – 0.19)*
Transitional Care Management	0	13.2	13.2	-	-	-
Office Visit	45.0	39.0	-6.0	0.11 (0.05 – 0.17)*	-0.22 (-0.25 – -0.19)*	-0.33 (-0.40 – -0.26)*
Specialist	44.3	48.3	4.0	0.07 (0.02 - 0.13)*	0.07 (0.02 - 0.12)*	-0.004 (-0.08 - 0.07)
Primary Care or Specialist	66.2	71.3	5.1	0.07 (0.004 - 0.13)*	0.14 (0.10 - 0.18)*	0.07 (-0.001 - 0.15)

© 2024 Anderson TS et al. *JAMA Health Forum.*

eTable 3: Interrupted Time Series Estimates of the Association between Transitional Care Management and 30-Day Post-Discharge Follow Up, by Demographics

	30-Day Primary Care Follow Up						
	2010, %	2019, %	Absolute Change, %	Pre-TCM Slope, quarterly % change (95% CI)	Post-TCM Slope, quarterly % change (95% CI)	Absolute Change in Slope, quarterly % change (95% CI)	Difference in Slope Change, quarterly % change (95% CI)
**Sex**							
Female	31.8	39.0	7.2	0.11 (0.05 - 0.18)*	0.23 (0.19 - 0.26) *	0.12 (0.04 - 0.19) *	-0.003 (-0.10 - 0.09)
Male	31.0	38.6	7.5	0.11 (0.06 - 0.16) *	0.23 (0.19 - 0.26) *	0.12 (0.06 - 0.18) *	Reference
**Race/Ethnicity**							
Asian	32.8	37.9	5.1	0.17 (0.10 - 0.24) *	0.14 (0.11 - 0.17) *	-0.02 (-0.09 - 0.04)	-0.09 (-0.60 - 0.42)
Black	25.6	31.4	5.8	0.13 (0.07 - 0.19) *	0.16 (0.13 - 0.19) *	0.03 (-0.04 - 0.10)	-0.10 (-0.20 - -0.004) *
Hispanic	30.7	35.4	4.7	0.10 (-0.02 - 0.21)	0.14 (0.10 - 0.17) *	0.04 (-0.07 - 0.15)	-0.09 (-0.22 - 0.04)
Other	32.7	38.5	5.7	0.08 (0.03 - 0.14) *	0.16 (0.13 - 0.20) *	0.08 (0.02 - 0.14) *	-0.05 (-0.14 - 0.04)
White	32.4	40.2	7.8	0.11 (0.06 - 0.17) *	0.24 (0.21 - 0.28) *	0.13 (0.06 - 0.20) *	Reference
**Rurality**							
Rural	33.5	42.2	8.7	0.08 (0.03 - 0.13) *	0.32 (0.26 - 0.38) *	0.24 (0.16 - 0.32) *	0.16 (0.06 - 0.27) *
Urban	30.9	37.8	7.0	0.12 (0.06 - 0.19) *	0.20 (0.17 - 0.23) *	0.08 (0.01 - 0.15) *	Reference
**Medical Dual Eligibility**							
Non-Dual	33.8	41.8	7.9	0.10 (0.03 - 0.17) *	0.25 (0.21 - 0.28) *	0.14 (0.07 - 0.22) *	Reference
Dual Eligible	26.8	31.9	5.0	0.14 (0.07 - 0.21) *	0.14 (0.10 - 0.18) *	0.004 (-0.08 - 0.09)	-0.14 (-0.25 - -0.03) *
**County-Level SDI Score**							
1-25	32.9	40.7	7.8	0.15 (0.10 - 0.21) *	0.24 (0.21 - 0.27) *	0.08 (0.02 - 0.15) *	Reference
26-50	32.5	40.4	7.9	0.13 (0.06 - 0.20) *	0.25 (0.21 - 0.29) *	0.12 (0.05 - 0.19) *	0.03 (-0.07 - 0.13)
51-75	31.3	39.4	8.1	0.11 (0.05 - 0.17) *	0.24 (0.20 - 0.27) *	0.13 (0.06 - 0.19) *	0.04 (-0.05 - 0.14)
76-100	29.0	33.7	4.7	0.04 (-0.02 - 0.10)	0.16 (0.13 - 0.19) *	0.11 (0.05 - 0.18) *	0.03 (-0.07 - 0.12)

© 2024 Anderson TS et al. *JAMA Health Forum.*

eFigure 1. Trends in 30-Day Post-Discharge Follow Up, 2010-2019

A) Primary Care

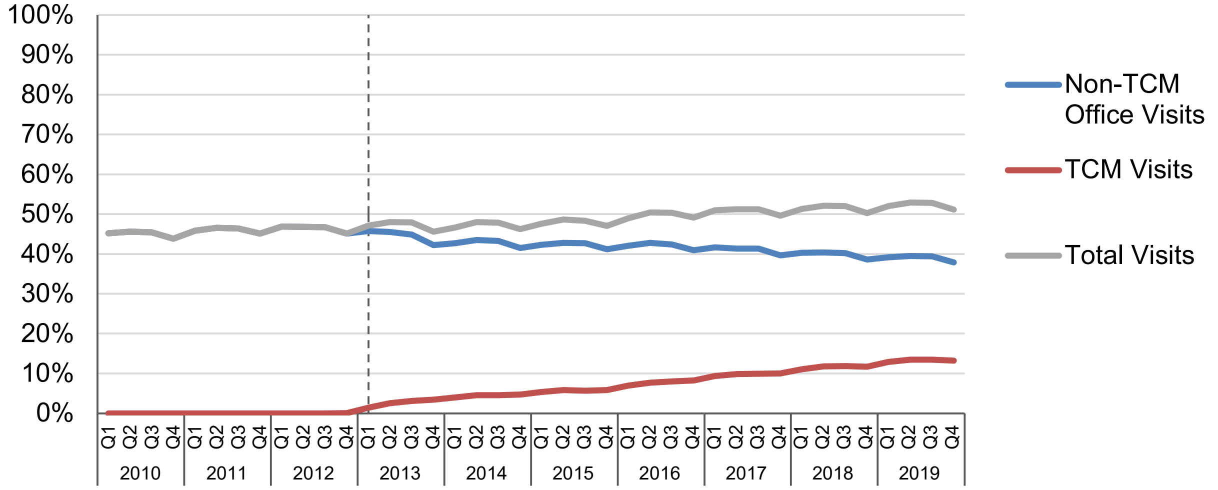

B) Specialty Care

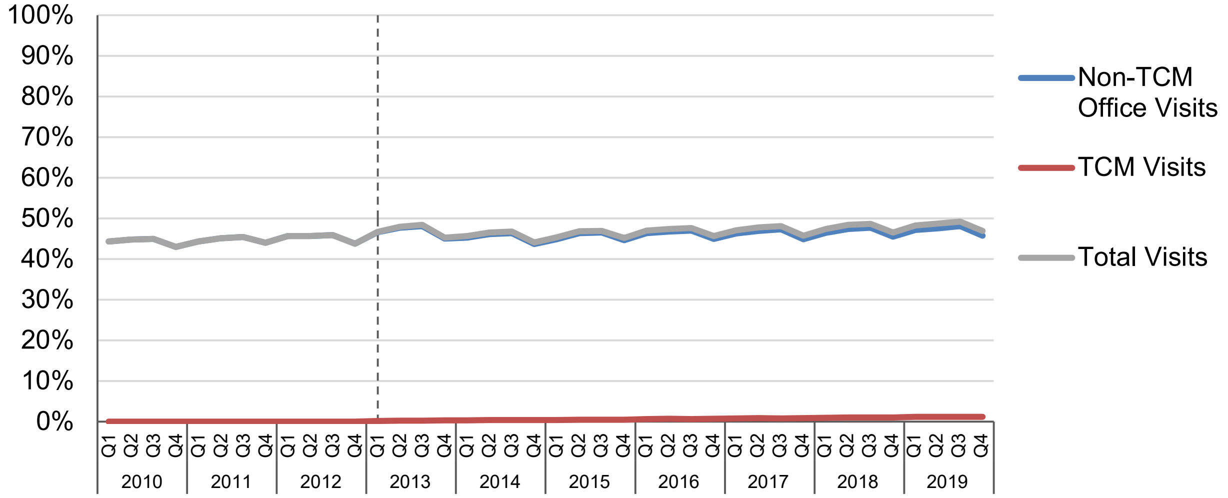

C) Any Follow-Up

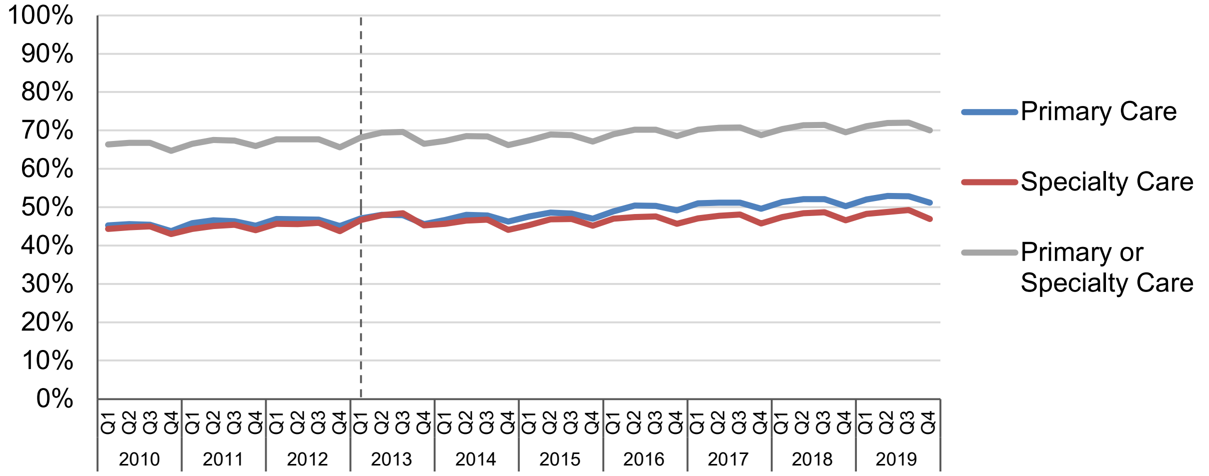

© 2024 Anderson TS et al. *JAMA Health Forum.*

TCM; Transitional Care Management

Note: Total refers to receipt of any ambulatory visit, TCM and/or non-TCM visits, within 30 days of discharge. Office visits refer to patients who received an office visit without a TCM billing code; patients who received both a TCM and non-TCM office visit within 30 days were grouped in the TCM category.

© 2024 Anderson TS et al. *JAMA Health Forum.*

eFigure 2: Rates of Transitional Care Management Visits in 2019, by Demographics

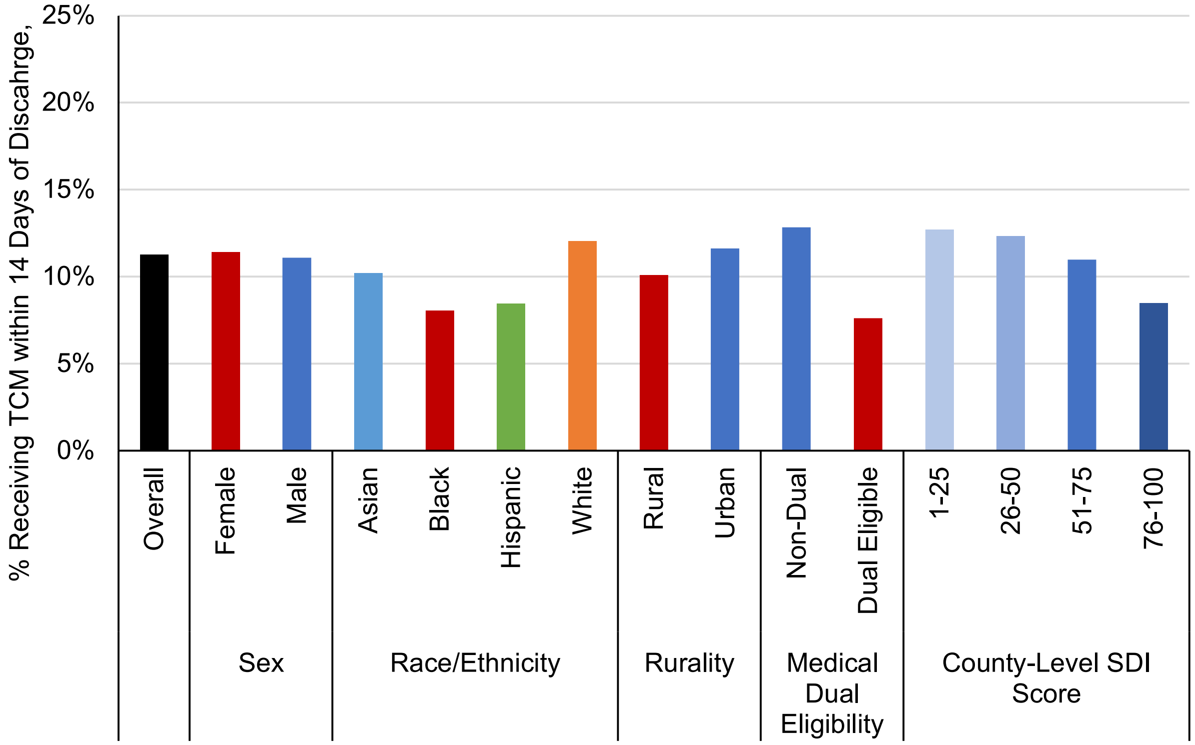

© 2024 Anderson TS et al. *JAMA Health Forum.*

eFigure 3. Trends in Timely Post-Discharge Follow Up, 2010-2019, by ACO Attribution

A) Primary Care

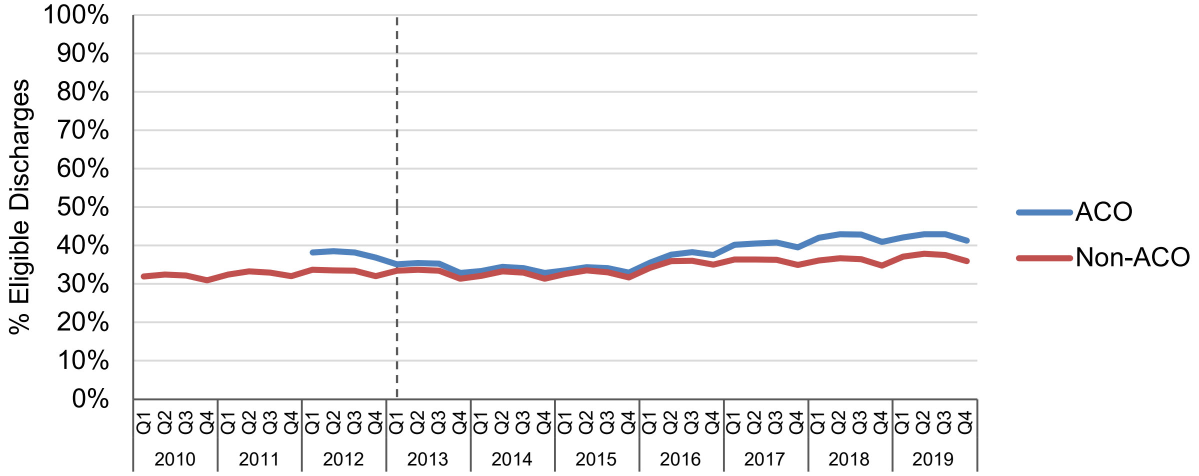

B) Specialty Care

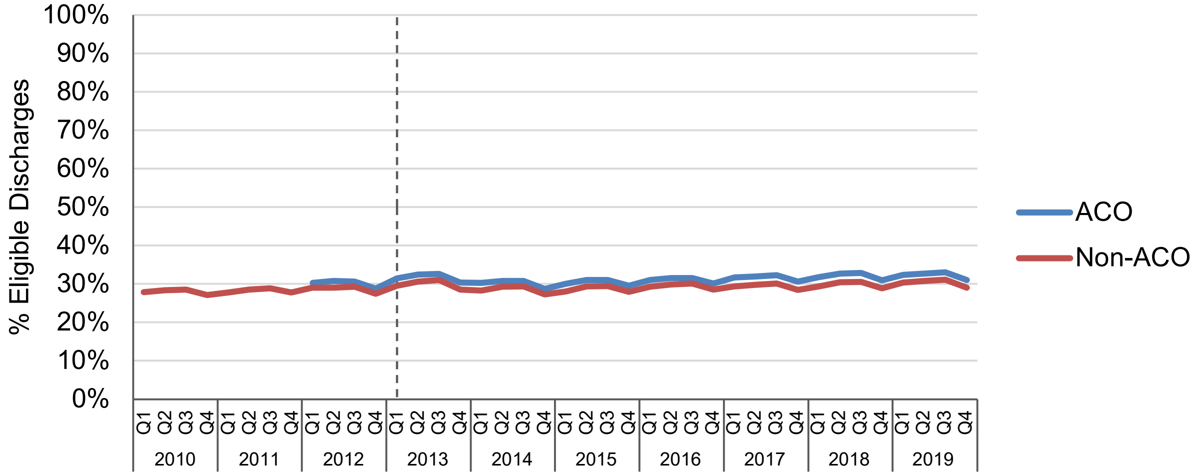

C) Transitional Care Management

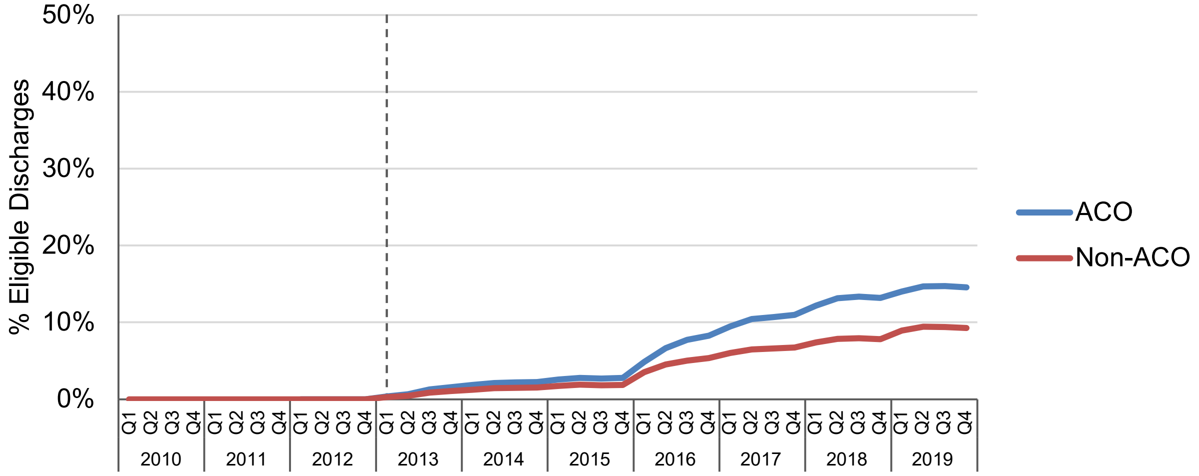

Note: Primary care and specialist visits refer to receipt of any ambulatory visit, TCM and/or non-TCM office visits, within 14 days of discharge.

© 2024 Anderson TS et al. *JAMA Health Forum.*

eFigure 4. Trends in Timely Post-Discharge Follow Up, 2010-2019, by Recent Primary Care

A) Primary Care

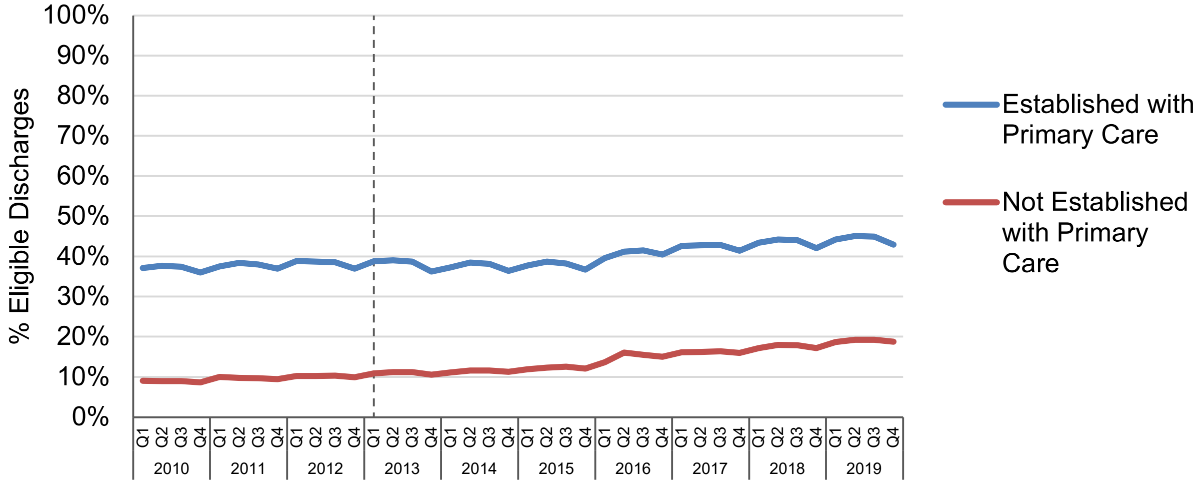

B) Specialty Care

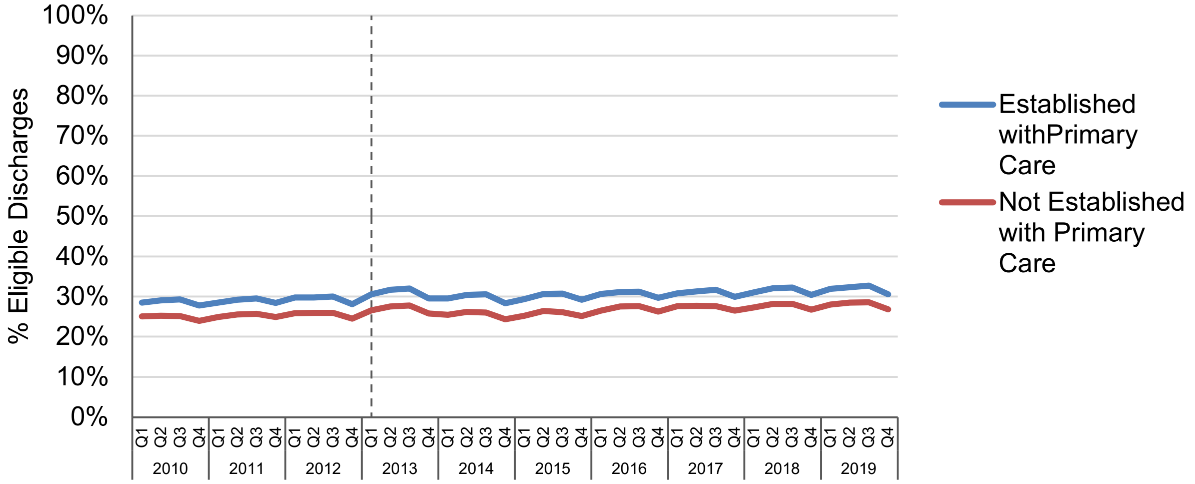

C) Transitional Care Management

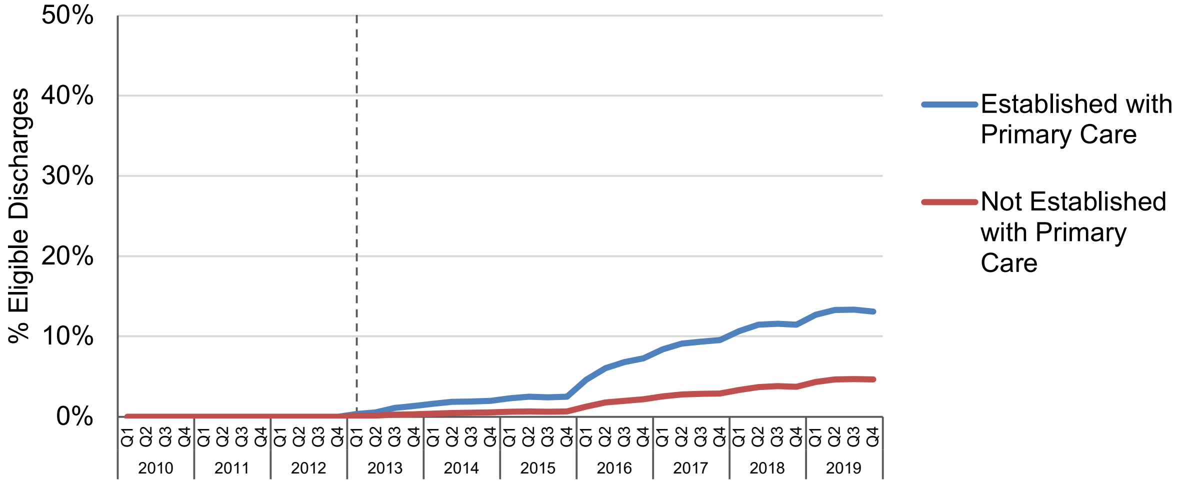

Note: Established PCP refers to having any primary care visit in the prior year. Primary care and specialist visits refer to receipt of any ambulatory visit, TCM and/or non-TCM office visits, within 14 days of discharge.

© 2024 Anderson TS et al. *JAMA Health Forum.*
