# Supplementary material for: Medicare Transitional Care Management Program and Changes in Timely Postdischarge Follow-Up
Source: JAMA Health Forum. 2024 Apr 12;5(4):e240417. doi: 10.1001/jamahealthforum.2024.0417 (PMC11065163; doi:10.1001/jamahealthforum.2024.0417)
Supplement: Supplement 2. — Data Sharing Statement [file jamahealthforum-e240417-s002.pdf]

Data Sharing Statement

Anderson. Medicare Transitional Care Management Program and Changes in Timely Postdischarge Follow-Up. *JAMA Health Forum*. Published April 12, 2024. doi:10.1001/jamahealthforum.2024.0417

Data
Data available: No

Additional Information
Explanation for why data not available: Medicare DUA agreement
